# Correction to “Acetate‐ Versus Lactate‐Buffered Crystalloids for Prevention of Post‐ERCP Pancreatitis in Patients Without Access to Rectal NSAIDs: A Multicentre Double‐Blind Randomized Trial”

**DOI:** 10.1002/ueg2.70294

**Published:** 2026-09-04

W. H. Paik, G. Huh, Y. H. Choi, et al., “Acetate‐ Versus Lactate‐Buffered Crystalloids for Prevention of Post‐ERCP Pancreatitis in Patients Without Access to Rectal NSAIDs: A Multicentre Double‐Blind Randomized Trial,” *United European Gastroenterology Journal* 14, no. 7 (2026): e70281, https://doi.org/10.1002/ueg2.70281.

In the author affiliations, affiliation 2 was published as “Department of Internal Medicine, Asan Medical Center, Seoul, Republic of Korea.” This should have read: “Department of Internal Medicine, University of Ulsan College of Medicine, Asan Medical Center, Seoul, Republic of Korea.”

We apologize for this error.

